# Digital Aids for Social Media in Adults With Traumatic Brain Injury: Longitudinal Evaluation Study

**DOI:** 10.2196/83639

**Published:** 2026-08-26

**Authors:** Dakota Sullivan, Nirav N Patel, Lisa Kakonge, Callie Y Kim, Yaxin Hu, Hailey L Johnson, Catalina L Toma, Melissa Duff, Lyn Turkstra, Bilge Mutlu

**Affiliations:** 1 Department of Computer Sciences University of Wisconsin–Madison Madison, WI United States; 2 Department of Hearing and Speech Sciences Vanderbilt University Medical Center Nashville, TN United States; 3 School of Rehabilitation Science McMaster University Hamilton, ON Canada

**Keywords:** accessibility, assistive technology, human factors, social media, TBI, traumatic brain injury

## Abstract

**Background:**

Digital social media aids are features that enhance accessibility to social media for users with disabilities. One group of adults with disabilities who have reported barriers to social media access is adults with traumatic brain injury (TBI). Results of previous studies showed that adults with TBI want to engage in social media but encounter significant barriers to doing so, due in large part to TBI-related cognitive and communication challenges. We previously collaborated with adults with TBI to develop 5 aids to increase social media access. Aids were implemented as Chrome extensions that modified user interface functions and visuals on Facebook. The present study was the next step: testing the use of the 5 aids by a novel group of adults with TBI longitudinally and in naturalistic settings.

**Objective:**

This study aimed to determine (1) how participants perceived each aid (ie, in terms of satisfaction, usability, likes, and dislikes), (2) how aids influenced participants’ well-being, and (3) which aid features participants rated as most and least useful.

**Methods:**

We recruited 39 adult Facebook users with TBI to take part in an approximately 1-month-long within-subject evaluation study of Facebook browser aids. Each aid was used for 3 days, followed by an evaluation and a subsequent 2-day no-aid use period. Participants evaluated each of the 5 unique browser aids in a randomized order.

**Results:**

Users reported the aids to be beneficial, especially aids that reduced visual clutter and information overload. Users also reported increased life satisfaction after using the aids (*P*=.02).

**Conclusions:**

These results provide proof of concept for the types of digital aids that could improve access to social media for users with TBI. We advocate including aids like these across all social media platforms so users with TBI can access the benefits of engagement in social media with fewer barriers.

## Introduction

### Overview

Moderate or severe traumatic brain injury (TBI) can cause cognitive and communicative challenges that profoundly affect a survivor’s activities of daily living and social networks [1-3]. Social media platforms have the potential to help individuals with TBI maintain and expand their social networks, join communities with people sharing similar experiences post injury, and acquire relevant information [4,5]. These platforms can also create spaces and opportunities for self-expression and identity construction post injury [6]. Individuals with TBI, however, report significant challenges when using social media platforms.

Building upon the existing literature on TBI-related social media challenges [6,7], our team designed and developed an accessibility toolkit to provide cognitive and communication support for individuals with TBI [8]. The toolkit includes 5 browser extensions that modify the Facebook interface for greater accessibility and usability. Here, we build on this prior work by testing how these tools perform during real-world, longitudinal use.

### Cognitive and Communication Impairments After TBI

TBI can cause a myriad of impairments in cognition, communication, and social interaction, as well as sensorimotor and psychological functions [1-3,9]. The most commonly impaired cognitive functions are executive functions and memory [2,10]. Executive function impairments can limit the individual’s ability to manage thoughts and feelings and self-regulate behaviors [11]. Memory impairments are typically in working memory, the ability to hold and manipulate information temporarily, and declarative learning, the ability to consciously encode new information. The combination of these impairments often leads to difficulties in higher-order cognitive functions, such as reasoning and decision-making [12,13].

Social cognition impairments have been well documented in adults with moderate to severe TBI, including deficits on affect recognition and Theory of Mind tasks [14-22]. While researchers continue to debate whether these impairments are secondary to impairments in executive function (eg, if impaired cognitive flexibility limits the ability of a person with TBI to take another person’s perspective) [23-25], there is consensus that performance on social cognition tasks is impaired and that these impairments contribute to negative social outcomes after TBI [26].

Cognitive impairments typically translate into communication challenges [25,27], including difficulty understanding long or complex verbal information, either written or spoken; failure to understand abstract or figurative language, like sarcasm or slang; and expressive language that is less informative, appropriate, efficient, or complete than would be expected in a given context [25,28]. Speed is also a factor in communication, as everyday communication requires rapid comprehension and expression. Speed of thinking and expressing thoughts is commonly affected by TBI [28], so even individuals with relatively strong cognitive function can have difficulty communicating at the pace of typical social interactions.

Use of inappropriate language and lack of social understanding can lead to limitations in family and friend support and cause social isolation and loneliness for individuals with TBI [1,29-33]. Psychological changes can also occur after TBI, causing negative self-perception, heightened stress and anxiety, and depressive symptoms [34,35]. Specifically, individuals with TBI can have a stronger emotional response to pessimistic information or negative experiences [8]. These TBI-related challenges can profoundly affect an individual’s social connections, resulting in a lower quality of life [36].

### Social Media Use After TBI

As a result of cognitive and communication impairments, individuals with TBI face significant barriers to using social media [4,8-42]. Complex user interfaces can be difficult to navigate and make some features of social media platforms less accessible [6,43]. Undesirable content in newsfeeds, such as arguments between users or posts pertaining to violence, can trigger heightened negative emotions and cause frustration for individuals with TBI, particularly individuals with impairments in emotion recognition. Advertisements in the newsfeed can easily be a source of distraction and prevent users from focusing on desirable content [8]. Because of their communication challenges, individuals with TBI can misunderstand social media posts, “misreading” the sentiment of the sender and thus responding with inappropriate comments [6,8]. These types of interactions can yield an overall worse user experience and limit the development of social connections [44].

### Why Improve Social Media Access?

It could be argued that the costs of social media access for adults with TBI outweigh the benefits, as cognitive challenges associated with TBI can pose risks to users. Our view is that adults with TBI who want to access social media should have the same opportunity to benefit from that access as their uninjured peers. When accessible, social media platforms help users maintain connections with their social ties, form bonds with communities, obtain new information, and foster information exchange [4,6,38,40,45]. For individuals with TBI, social media platforms provide functions that can help them overcome TBI-related challenges [4,5,38,43], and there is evidence that individuals with TBI may even prefer to have social interactions online rather than face-to-face [39]. Specifically, social media platforms can mitigate the communication challenges caused by TBI by reducing time pressure through increased time for users to comprehend information and formulate responses [4]. Individuals with TBI can also form online support groups to exchange information related to TBI and share personal experiences [4,5]. Moreover, individuals with TBI have used social media as a space to rebuild their self-identity and social connections post injury, particularly those who face dramatic changes in their personal lives [6]. With these benefits in mind, we next discuss our team’s prior work toward making social media more accessible to users with TBI.

### Related Work

#### Overview

There is a growing body of work aimed at reducing social media–related challenges faced by individuals with TBI and improving their social media experience [6,7]. For example, Brunner et al [46] developed online training and an educational toolkit for users with acquired brain injury to ensure users’ safety and privacy in social media use [46]. This important work has reduced barriers and risks to using social media but has focused on training persons with TBI rather than changing the platforms themselves. Our research team focused on the latter: developing tools that address these specific challenges and improve the social media experience for users with TBI.

Our research team sought to understand the challenges of social media use in users with TBI, with the long-term goal of reducing barriers to use and improving opportunities for communication, connection, and well-being through computer-mediated communication. In this section, we review our previous efforts toward the development and evaluation of social media aids, which have used a variety of research designs, including needs finding [5,39,47], participatory design [6], prototyping [48], and a formative study [8]. These studies culminated in our current longitudinal study assessing the effectiveness of the SMART-TBI extensions.

To begin, Zhao et al [48] conducted a preliminary study to assess individuals with TBI’s perceptions of the acceptability of 4 prototype social media aids intended to support user attention, memory, social cue interpretation, and message production. These 4 support areas were chosen based on existing literature and the clinical experience of the research team. Individuals with TBI were asked to review images of the prototype aids, read descriptions of their functions, and rate the aids in terms of acceptability, ease of use, and utility. The findings of this study indicated that participants found the proposed aids easy to use and expressed interest in utilizing them regularly. This study offered foundational insights for the future development of technological support tools on social media platforms.

Building on this work, Lim et al [6] further explored the use of social media supports through a participatory design study that involved exploration, ideation, and sketching to create prototype supports. Through this study, several critical design features were identified to address challenges faced by users with TBI on social media. These features included a safe filtering mechanism for the newsfeed, a customizable interface, and prompts to support communication, among many others. The study emphasized the importance of addressing not only sensory-level support but also cognitive, emotional, and communication impairments, along with the altered sense of identity experienced by users with TBI when using social media. The generation of these prototypes allowed the research team to ground future aid development in the needs of real users with TBI.

**Figure 1:**
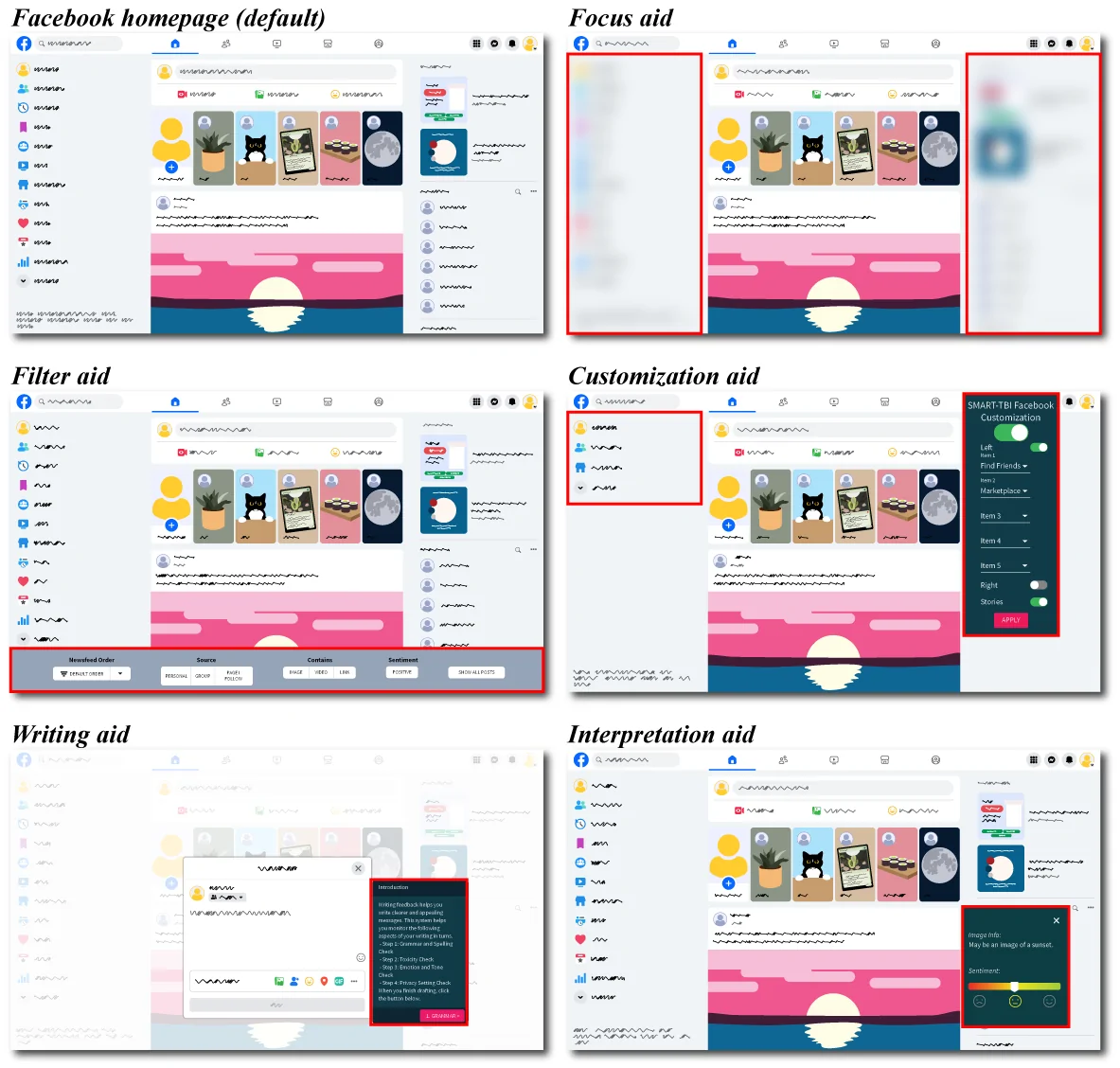
Social Media Accessibility and Rehabilitation Toolkit (SMART-TBI) extensions. Comparison of the default Facebook homepage and visualizations of each aid extension. Focus aid: the left and right side panels are blurred to increase focus on the newsfeed. Filter aid: a menu bar at the bottom of the interface allows users to tailor newsfeed content to their preferences. Customization aid: users can tailor the stories and the left and right side panels of the interface to meet their needs and preferences. Writing aid: users receive feedback on the posts they write, including grammar, spelling, toxicity, tone, and privacy checks. Interpretation aid: users are provided with an interpretation of a post’s content. The interface differences associated with each aid are highlighted in red.

Based on these participant-led designs, Hu et al [8] developed the Social Media Accessibility and Rehabilitation Toolkit (SMART-TBI). The toolkit consists of 5 aids: the focus aid, filter aid, customization aid, writing aid, and interpretation aid. These aids were specifically designed to address the cognitive and communication challenges faced by individuals with TBI on social media platforms. A visualization of each aid can be seen in Figure 1.

#### Focus Aid

This aid helps users focus on the newsfeed section of the Facebook interface by blurring other peripheral information present on the home page. This function reduces the cognitive challenge of information overload and creates a more manageable viewing experience. As a result, users with TBI are able to direct their attention toward meaningful content rather than extraneous details.

#### Filter Aid

Rather than limiting the amount of information on screen, this aid offers users the ability to reorganize newsfeed content in chronological order or view only positive posts. In doing so, this aid reduces cognitive challenges related to information overload and maintaining focus by allowing users to control the content they view and limit the appearance of undesirable content. IBM Watson NLP API [49] is used to analyze the sentiment of the posts and, in turn, enable positive post filtering.

#### Customization Aid

Similar to the focus aid, this aid helps users simplify the home page layout by offering the ability to customize which features are shown in the left column of Facebook’s home page (eg, friends, groups, and events), hide the right column (eg, advertisements and contacts), or hide the stories sections of the home page. While the focus aid also minimizes extraneous information on the home page, the customization aid enables users to optimize their navigation experience by choosing which elements they prefer to view. This aid helps to address cognitive challenges related to organization and planning, maintaining focus, and information overload.

#### Writing Aid

Unlike the prior 3 aids, this aid primarily focuses on communication by helping users refine their Facebook posts and comments through provided grammar suggestions, sentiment analysis (ie, tone and emotion type), toxicity analysis, and privacy settings (ie, private or public). This function allows users with TBI to better understand how others might interpret their posts and comments. As a result, this aid helps to address communication challenges related to social cognition, comprehension, word finding, and language production. Grammar errors, sentiment, and toxicity are detected using external APIs, including TextGears [50] for grammar checks, IBM Watson NLP API [49] for sentiment and emotion analysis, and Perspective API [51] for toxicity assessment. It is important to note that the writing aid runs entirely on the client side as a browser extension. To generate feedback, text is sent from the participant’s browser to the aforementioned natural language processing services. These services return only numeric scores and category labels. Google APIs are used under terms that prohibit data retention and training. We also set Perspective API calls so that text is not stored. TextGears stores only the analysis result and the number of API requests.

#### Interpretation Aid

While the writing aid helps users with TBI understand how others might interpret their posts and comments, the interpretation aid allows users to understand the sentiment and meaning of posts written by others. This aid helps to address the communication challenge of comprehension by highlighting negative text in red and positive text in green, thereby clarifying the sentiment of the post. The same external APIs used within the writing aid are also used to analyze the sentiment of posts in the interpretation aid (ie, IBM Watson NLP API [49]).

These 5 aids are intended to directly address the specific challenges faced by users with TBI. As a result, we hope to see improvements in participant self-reports, including feelings of emotional support, social connection, self-esteem, and satisfaction with life.

### Objective

We conducted a longitudinal in-the-wild study to evaluate the effectiveness of the 5 aids described above in supporting users’ social and psychological well-being. A total of 39 users with TBI engaged in this study over the course of 4 to 7 weeks. Throughout the course of the study, all participants completed a prestudy interview, provided daily numerical and open-ended ratings of their satisfaction with each aid, completed evaluation surveys of each aid after each evaluation period, and completed a poststudy interview describing their experience throughout the study. Through these data, we sought to answer the following questions:

- How do participants perceive each of our 5 SMART-TBI extensions in terms of satisfaction, usability, likes, and dislikes?
- How do the aid extensions influence participants’ well-being?
- What aid features are most and least useful to participants?

The contributions of this work are threefold:

- a longitudinal evaluation of our SMART-TBI extensions in participants’ everyday use;
- insights into the social media experience of users with TBI, including their challenges and needs; and
- design implications for social media developers who may integrate the features of these aids into their applications.

## Methods

### Overview

We next describe our approach to evaluating the SMART-TBI extensions, including details on participants; measures; study materials, design, and procedure; and our analysis plan.

### Participants

A total of 39 adults with moderate to severe TBI (female: n=25; male: n=14) were recruited at Vanderbilt University Medical Center (n=30) and McMaster University (n=9). At the time of the study, the participants’ mean age was 41.85 (SD 11.55) years. All participants were from the continental United States and were native speakers of North American English. While some participants occasionally missed their daily Facebook usage, all 39 participants successfully completed all 5 evaluation phases of the study for a final retention rate of 100%.

Participants at the Vanderbilt site were recruited through the Vanderbilt Brain Injury Patient Registry [52], and participants at the McMaster site were recruited from among participants in previous studies who consented to be recontacted for future studies. All participants with TBI were in the chronic phase of injury (ie, >6 months post injury). All but 1 participant sustained their injuries in adulthood (ie, at or after age 18 years), and the average time since injury among participants was 8 (SD 8.37) years. Participants did not have a history of neurological or cognitive disabilities before the qualifying brain injury. TBI severity was determined using the Mayo Classification System [53]. By this system, participants were classified as having sustained a moderate to severe TBI if at least one of the following criteria was met: (1) a Glasgow Coma Scale (GCS) score of less than 13 within 24 hours of acute care admission, indicating moderate or severe injury; (2) positive neuroimaging findings; (3) loss of consciousness (LOC) greater than 30 minutes; or (4) posttraumatic amnesia (PTA) after 24 hours. Injury-related information was collected from available medical records and a semistructured interview with participants. GCS scores were available for 26 participants, with scores ranging from 3 to 15; LOC was available for 28 participants; and PTA information was available for 29 participants. Acute imaging information was available for 27 participants, with 26 containing positive findings. Causes of injury included motor vehicle accidents (n=16), falls (n=9), motorcycle accidents (n=6), being hit by a car as a pedestrian (n=3), assault (n=2), multiple concussions (n=1), or other (n=2). Multimedia Appendix 1 provides demographic and injury characteristics of our participant population.

Inclusion criteria for this study included self-reported English fluency, having an active Facebook account and using it within the past year, knowing the login information for the account, and daily access to Wi-Fi.

### Study Materials

The research team provided each participant with a Samsung Chromebook 4 laptop (model #XE310XBA-K01US), which was used for all study-related activities (eg, Facebook usage and Zoom meetings). To prevent lack of familiarity with a Chromebook from being a barrier to participation, the research team prepared each Chromebook with a new Gmail account, Zoom for aid activation and prestudy or poststudy calls, all 5 SMART-TBI extensions, and aid demonstration videos for each aid. Additionally, each laptop contained browser bookmarks for Facebook and other relevant applications (eg, YouTube and Gmail) required throughout the study. Each participant also received a binder with their personalized schedule, a series of “cheat sheets” describing the functions of each aid, and login information for both the Chromebook and Gmail account. Each participant was also provided with a wireless mouse and keyboard for easier navigation of their laptop and a laptop bag to hold all the study materials.

### Study Design

This study used a within-subjects, in-the-wild design. The study protocol was approved by the institutional review boards of Vanderbilt University and McMaster University, and all participants provided consent prior to engaging in the study.

**Figure 2:**
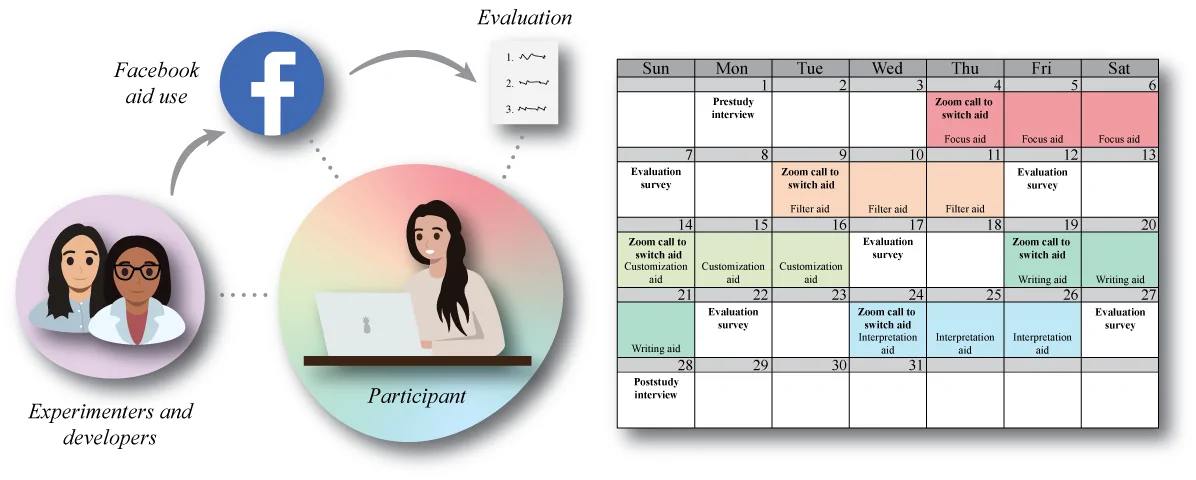
Participant schedules. Participants engaged in the study following schedules with the same structure illustrated here. Participants initially met with experimenters to complete a prestudy interview and later met with experimenters at the beginning of each aid evaluation period to activate the relevant aid. Each evaluation period consisted of 3 days during which participants used 1 of the aid extensions. Following each evaluation period, participants completed an aid evaluation survey. After all 5 evaluation periods, participants completed a final poststudy survey. Participants additionally met with the development team to discuss any technical challenges as needed throughout the study.

Participants used the developed aid extensions for 4 to 7 weeks, with the duration of a given participant depending on external factors such as travel and holiday plans. Each participant received a personalized study schedule tailored to their individual availability and preferences (eg, time of day for Zoom calls). An example schedule is shown in Figure 2.

To begin, participants attended an in-person or online prestudy session where they were introduced to the study and provided with study materials. Throughout the course of this study, participants were asked to use and evaluate the 5 SMART-TBI extensions. Each evaluation included 3 days of using an individual aid, followed by a period of Facebook use with no aids to mitigate carryover effects. The number of baseline days varied depending on individual schedules and availability, but participants were always given at least 2 days between each aid evaluation phase. The aid extension order was randomized using a balanced Latin-square design to reduce order effects. At the beginning of each evaluation phase, participants were required to complete a Zoom call to activate the necessary aid for that period. After using all 5 aids, participants attended an online poststudy session to end the study.

Participants were compensated US $20 per hour for the prestudy session, poststudy session, and each of the 5 online aid introduction sessions. For the duration of the study, participants received US $10 per day that they used Facebook for at least 10 minutes. Compensation was provided in the form of a check or Amazon e-gift card. Finally, participants who completed the study were gifted the Chromebook laptop they used throughout the study.

### Procedure

#### Prestudy Session

Participants were given the option of completing the prestudy session online or in person. If a participant chose the in-person option, the research team met the participant in the laboratory and gave them the Chromebook laptop to take home for the rest of the study. If a participant chose the online option, the research team mailed them their Chromebook laptop and met with them on Zoom. During the prestudy session, participants were given an overview of the Chromebook (ie, login information and bookmarks) and their study schedule (ie, evaluation days, baseline days, and survey dates). Prior to or during this session, participants provided informed consent by signing digital consent forms.

Once a participant logged into their assigned Chromebook, they completed a prestudy interview on REDCap and a prestudy questionnaire on Qualtrics. Next, participants logged into their Facebook accounts and were informed that their Facebook usage would only register if they used the provided Chromebook. Additionally, the experimenters informed participants that data collected throughout the study would never include the specific content of their posts and would be anonymized through the use of participant IDs.

Following this initial meeting, participants began the study by completing baseline days (ie, using Facebook with no aids active) until the first scheduled Zoom meeting, during which the first aid would be activated and the evaluation phase would begin.

#### Aid Evaluation Phases

Throughout the course of the study, participants completed a total of 5 aid evaluation phases. Each phase began with a Zoom call with an experimenter to (1) update the laptop background to the “cheat sheet” that matched the aid being evaluated, (2) assist the participant in activating the aid and a “helper window” extension for feedback, (3) watch the demonstration video for the relevant aid, and (4) guide participants through the process of using the aid prior to individual use. Participants were asked to use the aid for 3 days and for a minimum of 10 minutes per day. Outside of the minimum usage time, participants were encouraged to use Facebook as they would during normal use. Following their daily use of Facebook, participants were asked to complete a brief feedback survey to report their satisfaction with the active aid. This survey was made available through a “helper window” extension. After 3 days of use, the active aid extension automatically deactivated. Participants were then sent an email with an evaluation survey to provide detailed feedback on the aid extension they had evaluated. This full process was then repeated for each of the remaining aids until all had been evaluated.

#### Poststudy Session

Following their evaluation of all 5 aid extensions, participants completed an online poststudy session with an experimenter. During this session, participants completed an interview on REDCap and a questionnaire on Qualtrics to report their experiences with the aids and well-being outcomes. Additionally, experimenters assisted participants in deleting study materials and restoring the laptop to factory settings during this time.

### Measures

#### Overview

Throughout the course of this study, the research team collected a considerable amount of data ranging from qualitative interviews and open-ended survey questions to quantitative perception measures and Facebook usage data.

#### Prestudy Interview

Within this initial interview, experimenters asked participants a series of 8 open-ended questions, which centered on current social media usage and experience with assistive technology for TBI challenges. A listing of these questions is provided in Multimedia Appendix 2.

#### Aid Evaluation Surveys

Following each aid evaluation phase, participants completed a survey concerning their experiences with and perceptions of the evaluated aid. This survey included the full 15-item Usefulness, Satisfaction, and Ease of Use (USE) Questionnaire [54], which captures perceptions of usefulness, ease of use, ease of learning, and satisfaction. This questionnaire is a widely used metric to evaluate system usability and was chosen to capture a multifaceted perspective on participant experience with the developed aids. Five open-ended questions concerning likes, dislikes, technical difficulties, aid challenges, and design suggestions were also included. These items allowed the research team to develop a thorough understanding of participant perceptions and experiences while also providing an opportunity for participants to give open-ended feedback. All items that were developed by the research team are provided in Multimedia Appendix 2.

#### Daily Ratings

After a participant had used Facebook on a given day, they completed a daily rating. Participants were prompted with the question, “How satisfied are you with the Facebook aid today?” followed by the optional question, “Do you have any other comments?” The first question was answered with a star rating of 1 to 5, while the second question was answered with a free-text response.

#### Poststudy Interview

During the final interview, experimenters asked participants a series of 3 open-ended questions centering on their experiences with the aids throughout the study. These questions included:

- How was your experience using the aids?
- Do you have a favorite aid? And if so, which one was it? And why?
- Is there anything else you’d like to share with us?

#### Prestudy and Poststudy Surveys

Within both the prestudy and poststudy surveys, participants rated their feelings of emotional support, social connection (adapted from Ledbetter [55]), self-esteem [56], and satisfaction with life [57]. These ratings were intended to capture any pre-post study changes. Additionally, the poststudy survey also contained 2 additional items regarding unaddressed TBI challenges (ie, “Are there any challenges related to your TBI that were not addressed by the aids you used during this study? If yes, please explain.”) and endorsements of the aids for use in everyday life (ie, “Would you use any of the aids you have used during this study in your daily life? If so, which ones would you use?”). After having evaluated all aids, these questions allowed the research team to understand the main benefits and shortcomings of all aids holistically.

### Analysis

Given the complexity of this study and the high volume of data collected throughout, quantitative data were first reviewed and cleaned to remove any erroneous data (ie, duplicate surveys and extraneous daily ratings). Following this step, aggregate daily ratings were generated for each aid per participant and then for each aid across all participants. Aggregates were also generated for each perception rating collected within the evaluation surveys across all participants. Finally, paired-samples 1-tailed *t* tests were used to determine whether there were positive pre-post changes in participant feelings of emotional support, social connection, self-esteem, and satisfaction with life.

We examined several types of qualitative data, including prestudy and poststudy REDCap interview data and multiple ratings of participants’ perceptions of the extensions (Table 1). We conducted inductive thematic analysis for the survey feedback under each category for each aid (ie, likes, dislikes, technical difficulties, and aid challenges) [58,59]. The primary coder and a secondary coder each coded a subset of the dataset and developed the initial codebook. Disagreements were resolved through discussion. The primary coder then coded the remaining dataset, consolidated new codes, and updated the codebook iteratively. The primary coder performed the interview analysis independently. This analysis was aided by qualitative analysis software, specifically Dedoose [60], that facilitated data management and coding consistency. Based on the final codebooks of the survey responses and the interview data, our interdisciplinary research team discussed and generated the final themes and subthemes.

**Table 1:** Data analysis approach. An overview of the analysis for all data collected throughout the longitudinal user study, including collection method, measures, and analysis approach.

Collection method	Measures	Analysis approach
Prestudy interview	Prior social media usage and experience with assistive technology for TBI^a^ challenges	Thematic analysis
Prestudy survey	Feelings of emotional support, social connection, self-esteem, and satisfaction with life	Descriptive statistics
Daily rating	Satisfaction with the evaluated aid	Descriptive statistics
Aid evaluation survey	Quantitative perceptions (ie, usefulness, ease of use, ease of learning, and satisfaction) and qualitative perceptions (ie, likes, dislikes, technical difficulties, aid challenges, and design suggestions) of aid extensions	Descriptive statistics and open coding
Poststudy interview	Experiences with all aids throughout the study	Thematic analysis
Poststudy survey	Feelings of emotional support, social connection, self-esteem, and satisfaction with life as well as aid endorsements	Descriptive statistics and *t* test

^a^TBI: traumatic brain injury.

### Ethical Considerations

The study protocol was approved by the institutional review board (IRB) at Vanderbilt University (IRB 191489) and the Hamilton Integrated Research Ethics Board at McMaster University (Project 16271). All participants provided informed consent prior to participating. Participants were compensated at a rate of US $20 per hour for the prestudy session, poststudy session, and each of the 5 online aid introduction sessions. During the study period, they also received US $10 for each day they used Facebook for at least 10 minutes. Compensation was provided by check or Amazon e-gift card. In addition, participants who completed the study were permitted to keep the Chromebook laptop used throughout the study. Participant data were deidentified through the use of codes. All identifying information present in interview data was removed during the transcription process. Additionally, all of our social media aids ran on the client side as browser extensions, and no content from any Facebook posts or comments was collected. Collected data, such as consent forms and electronic data files, were stored in either secure physical storage or university-approved servers accessible only to study personnel. In the case of Vanderbilt University, research records will be retained for at least 3 years following closure of the research with the IRB, and any HIPAA (Health Insurance Portability and Accountability Act)-related documentation will be retained for at least 6 years following the last date of use. Deidentified data collected at McMaster University will be stored indefinitely.

## Results

### Overview

In this section, we present the results of our longitudinal study, including themes derived from our prestudy and poststudy interviews, findings from our multifaceted aid evaluation, and the overall impact the aids had on participants.

### Prestudy Interview

#### Overview

To begin, a total of 39 prestudy interview transcripts were analyzed using thematic analysis. Through this analysis, we identified a total of 6 themes that illustrate the varying backgrounds of our participants.

#### Theme 1: Facebook Usage Changes Following TBI

##### Overview

Our analysis revealed several subthemes that reflected reported changes in patterns of Facebook usage in relation to TBI-related challenges.

##### Pattern of Usage

A total of 51% (20/39) of participants noted a decrease in Facebook use following their TBI, with varying patterns of reduced engagement. Some participants ended their use immediately after their TBI and only re-engaged in a limited manner sometime later. Participant 5 stated:

I just got back on Facebook about a year and a half ago. If I added anyone, it was just immediate family, a pool of ten people. These people don’t have Instagram, so I have them on Facebook...I rejoined because I missed seeing the progression of close people’s stories.

Others reported decreasing or ending specific activities, such as posting. For example, participant 27 explained:

I have stopped posting a ton of things on Facebook. I limit it to just family things and not so much on where I am, like checking in anywhere.

Some participants expressed a preference for re-engaging with visual-oriented platforms, such as Instagram, due to their TBI-related needs or personal interests. Participant 1 mentioned, “I switched over mostly to Instagram, with it being easier to take a look at pictures.” Additionally, several participants indicated a desire to limit their time online or adapt their social media use to better suit their post-TBI lifestyle. For example, participant 10 noted:

Not specifically due to my TBI, but I do use social media significantly less than I did before. Now I primarily use Facebook for Facebook Marketplace and to remember birthdays.

Participants 1 and 5 also noted a switch to Instagram from Facebook post TBI, as the picture-based nature of Instagram supports their engagement.

However, 10% (4/39) of participants noted an increase in their Facebook use post TBI. Participant 18 remarked, “My Facebook use has increased since the injury, partially due to COVID since I was home more.” This participant also attributed their increased use to social pressure, stating:

It has increased due to social norms forcing the use of Facebook to be able to be involved in community events, etc. since that is how they notify you of times and changes in events.

Additionally, 31% (12/39) of participants did not report any specific changes in their Facebook usage post TBI. For example, one participant noted that they “use it about the same” and described themself as “not a frequent user” (P31).

##### Sensory Challenges

Additionally, 21% (8/39) of participants noted sensory challenges while engaging with Facebook post TBI. Specifically, vision challenges were reported to impede Facebook navigation immediately following a sustained TBI:

The biological part, my eyes were so bothered by the bright light. Screens were just out, completely. Scrolling was impossible. I would close my eyes as I was scrolling and then look at posts, it was a nightmare. Trying to read people’s updates, I found it was frustrating and a useless experience.

P5

Participants also described experiencing sensory overload while on Facebook, noting, “It just goes very fast; it takes me a while to understand” (P4).

##### Social Challenges

Further, 10% (4/39) of participants noted social challenges while engaging on Facebook post TBI. One such challenge was reduced confidence in posting on Facebook:

Actually, before I was confident using it. Now, I will start to put something on Facebook and then delete it because I’m not confident about what I’ve posted. It’s wording, punctuation, that I’m not as confident with.

P7

Concerns with social comparison were also expressed: “I had deleted Facebook initially. I couldn’t watch everyone’s updates of getting married, having kids” (P5). This sentiment was echoed by other participants who felt overwhelmed by others’ lives:

The way I see Facebook, it’s more attracting families, moms and dads, posting their pictures—blasting all of their pictures, like birthday parties—It’s too much.

P1

##### Cognitive Challenges

Beyond changes in Facebook usage, many participants also noted challenges following their TBI. Changes in cognitive abilities, such as attention, memory, and understanding, were specifically noted by 8% (3/39) of participants. They shared that their “attention span” was reduced when on Facebook (P33), that they relied on written reminders to support their memory (P6), and that understanding online information was difficult at times: “Some things I don't understand. Definitely. It’s difficult sometimes” (P4).

#### Theme 2: Use of Technology, Apps, and Tools for TBI Management

##### Overview

Participants described the use of various tools, apps, and technology to manage their TBI symptoms in general and specifically to support their use of online communication. The following subthemes relate to the types of supports participants indicated they used prestudy.

##### Cognitive Supports

A total of 26% (10/39) of participants highlighted the use of various technologies and apps to address cognitive challenges following a TBI, both in their daily lives and on Facebook, where available. These tools were primarily used to support memory and attention difficulties. For instance, participant 3 mentioned:

I’ve attempted to use a lot of apps for research on symptom management, for example like a type of exercise to do to overcome the dizziness, the cognitive impairment aspect of it, and the balance of course.

The types of tools and apps used included timers, calendars, reminders, alarms, note-taking apps, and meditation apps. These cognitive supports were considered essential by many participants. One participant emphasized the importance of these supports, stating, “It’s kinda’ like my external hard drive. When I can’t rely on my brain, it’s helpful for storing and recalling information” (P8). Participants also discussed the use of various cognitive retraining apps, including brain games and specific apps (eg, Peak) “to improve brain function and stamina” (P12).

Participants also noted challenges with the consistent updates to and availability of these tools on social media. For example, participant 10 expressed difficulty in keeping up with changes on social media platforms: “I also like the birthday reminders but don’t currently know where to find this on Facebook; things seem to move often.”

##### Sensory Supports

A total of 21% (8/39) of participants discussed the types of apps, tools, and technology they use online to cope with visual, sensory overload, and hearing challenges. Visual supports included setting their screen to dark mode, dimming the screen, using a larger font, and orienting the screen to photo mode. Participant 19 noted, “I like darker screens now and less clutter, so we’ve adapted my preferences to be darker and less clutter[ed]; email, my search engine, etc.” Similarly, participant 5 stated, “I use larger font—14-16 size font,” to support their vision. Additionally, one participant mentioned post-TBI auditory sensory overload and the use of “hearing aids...for anacusis and tinnitus, with active noise canceling” (P5).

##### Assistive Technology

Additionally, 21% (8/39) of participants described an increased reliance on the use of assistive technology in the form of devices and assistive features post TBI. For example, some described using larger devices to access social media: “My iPad and computer have purposefully lots of pixels, big screens, etc., for when my eyes get tired and it’s harder to process” (P19). Specific assistive features noted included speech-to-text, text-to-speech, and AssistiveTouch. Reasons participants reported for using these features centered around the need for cognitive, physical, and communication supports. For example, one participant noted:

With the voice-to-text, because of the affected side [of my body], sometimes it is hard for me to write or type. So I use the voice-to-text. It also helps my speech because the injury affected my speech. In the beginning, you get a lot of therapy but then it stops. I also use the touch screen and magnifying and closed captioning a lot.

P3

##### Written Communication Supports

Some participants (3/39, 8%) also reported using spelling and grammar check tools while writing online, specifically highlighting the use of apps like Grammarly to support post-TBI cognitive challenges with writing. Participant 36 shared:

When I forget a word or erroneously substitute, [for example I] meant ‘baseball’ but typed ‘basement’, I like that it highlights potential errors and allows me to decide if I want to ‘correct’ it.

sic

##### AI

AI search tools, such as Bard AI, were described by one participant as “helpful when sifting through large amounts of data” (P2) and were highlighted for their role in facilitating information processing and retrieval post TBI.

##### Lack of Support

A substantial number of participants (16/39, 41%) reported not using any specific apps or technology post TBI. Participants’ rationale varied, ranging from a lack of awareness about available tools to a preference for managing their interactions with technology in other ways. For instance, participant 18 stated, “[I do] not know of any tools to use,” indicating a lack of awareness as a barrier to using supportive technology. Some participants preferred to manage their technology use by controlling their exposure to overwhelming stimuli. For example, participant 27 shared their strategy for handling Facebook usage: “I got back onto Facebook once I could handle it again. If I became overwhelmed using it, I would stop and come back to it later.” This approach highlights a preference for self-regulation and intermittent use to avoid sensory and cognitive overload.

#### Theme 3: Frequent Use of Social Media and Facebook

Out of 39 direct responses regarding the frequency of social media and Facebook use, 37 (95%) participants provided detailed insights. The majority of participants (34/39, 87%) rated their use as “5,” indicating they use social media “very frequently” (eg, daily or several times a day). Example comments included: “[I] check stuff daily but more app-specific preferences” (P36). Some participants noted that they are on social media “constantly, I’m on it all the time” (P2), or “on a daily basis, 2-3 hours per day, this is where I get my news now” (P1). A total of 2 (5%) participants rated their use as “4,” signifying that they use social media often (eg, once a week to several times a week). One participant rated their use as “2,” indicating “rare” use of social media (eg, less than once a month), stating:

Maybe once a month or once every two months [I use Facebook]. Then I’m only on there for a few minutes to send a message or receive one. I find it overwhelming and confusing and too time consuming for me.

P19

As with the frequency of social media use, the majority of participants noted that they use Facebook either “very frequently” (27/39, 69%) or “often” (8/39, 21%). One participant (P35) noted that they use Facebook “rarely,” specifically “once every 3 months.” Participants discussed attempting to limit their use, noting, “[I am on] daily but [for a] limited amount of time; mostly to check on community events, not for personal people [sic] networking” (P18). Another participant mentioned a specific use of Facebook to keep up with a certain demographic of friends:

I check it once a week primarily to keep in contact with older sergeants from my time in the Army that only use Facebook, along with some members of my family.

P23

#### Theme 4: Preferred Social Media Platforms

Participants described using multiple social media platforms in addition to Facebook, with many engaging with 2 or 3 other platforms. Instagram was mentioned most frequently (30/39, 77%), followed by TikTok (9/39, 23%), Snapchat (5/39, 13%), and several others. A visual of the reported social media platforms is provided in Multimedia Appendix 3.

One participant explained their selective use of social media platforms:

I was on Instagram, but I don’t like it. I just find that everything is about your picture and posting, and I’m getting overwhelmed with social media. So, I just choose which one is functional for me. And TikTok, but it’s getting too addictive, I like the dancing and the funny stuff. Of course, YouTube—everything you need is on YouTube. And YouTube is educational for me. I like fixing stuff so I look for how to do stuff.

P3

Several participants (9/39, 23%) noted that Facebook was the only social media platform they actively used. One participant shared:

Facebook’s the only one I use. I Google things and use email, but that’s about it. I don’t have Instagram or TikTok or Twitter. If there are other social media platforms, I’m not really aware of them...

P19

#### Theme 5: Perceived Benefits of Social Media

##### Overview

Participants were asked what they generally liked about engaging on social media, specifically on Facebook. Several subthemes of liked aspects of social media emerged.

##### Social Connection

Most participants (32/39, 82%) reported that engaging on social media provided them with an important context for social connection. They reported using social media to connect with friends and family who live far away, converse with new people with similar interests, and stay updated with others’ activities. One participant noted about Facebook:

After my brain injury, I like the fact that I can, it can be two in the morning or afternoon, there’s always somebody, a conversation, something to see. It’s very stimulating.

P9

##### General Information

A total of 26% (10/39) of participants highlighted the ease of accessing information, such as staying current with the news and learning new things. For example, one participant mentioned, “It’s great for staying up to date with the news” (P24), and another added, “I use it for learning new things” (P21).

##### Entertainment

Several participants (6/39, 15%) described the entertainment value of watching videos, sharing memes, and passing time with humor. One participant noted, “I follow very few things on Facebook, but the ones that I do are fun things” (P7).

##### Professional Development

Some participants (3/39, 8%) said they used specific sites for business purposes. One participant said that LinkedIn was a “powerful tool in the business environment” (P23), allowing them to build their businesses.

#### Theme 6: Concerns With Facebook

##### Overview

While about half of the participants (18/39, 46%) reported not having any concerns with their Facebook use, the others described concerns about Facebook in 4 categories: cognitive, social, privacy and safety, and skills-related.

##### Cognition

Cognitive concerns included feeling overloaded and overwhelmed by information and concerns about their own overuse of the platform. For example, participant 31 noted, “It takes up too much time, too much screen time, [and] feels like it makes my attention span worse.” Another participant echoed this sentiment, stating, “I have ADHD and it’s a dopamine hit, so I’m prone to get on social media too much. Especially TikTok” (P21). One participant reported concerns with their long-term use of Facebook, noting, “my brain has enough challenges, and it’s hard to keep up with all the changes on Facebook” (P19).

##### Social

Social concerns included personal and social judgments about what they and others posted, as well as social comparison. Participant 1 said, “Sometimes I feel weird about posting and people asking too much [sic] questions,” and participant 30 expressed the concern that Facebook “leads to comparison of others.”

##### Privacy and Safety

Privacy and safety concerns included concerns about data tracking, identity and information theft, scammers, hacking, and a lack of anonymity. For example, participants reported knowing their information is not private and feeling “leery” about posting or experiencing heightened anxiety online. Participants noted, “There are so many unscrupulous people” (P2) and “Predatory people are out there, and [you] need to be careful” (P9).

##### Lack of Skills

Finally, skills-related concerns centered around familiarity with using Facebook. For example, participant 7 explained why they chose Facebook because of its familiarity:

Because I’m used to Facebook, that’s why I go there the most. Others, like Instagram, I’m not used to moving around, navigating. With Facebook, I get a pop-up message that it’s there.

Other participants expressed concerns with frequent Facebook interface updates and the resulting reduced confidence with the platform. These reports showcase the importance of consistency in allowing for a manageable social media experience.

### Aid Perceptions

#### Overview

To understand how participants perceived each of our aid extensions, we investigated their daily ratings (ie, numerical and descriptive) while the aids were active and their perceptions of each aid holistically across the metrics of the USE Questionnaire (ie, usefulness, ease of use, ease of learning, and satisfaction). We also conducted open coding of participants’ reported likes and dislikes, technical difficulties, and aid-specific challenges for each aid. Finally, we analyzed which aids participants reported they would like to continue using in daily life. These data allow us to understand which aid features were beneficial and where improvements can be made.

#### Daily Ratings

**Figure 3:**
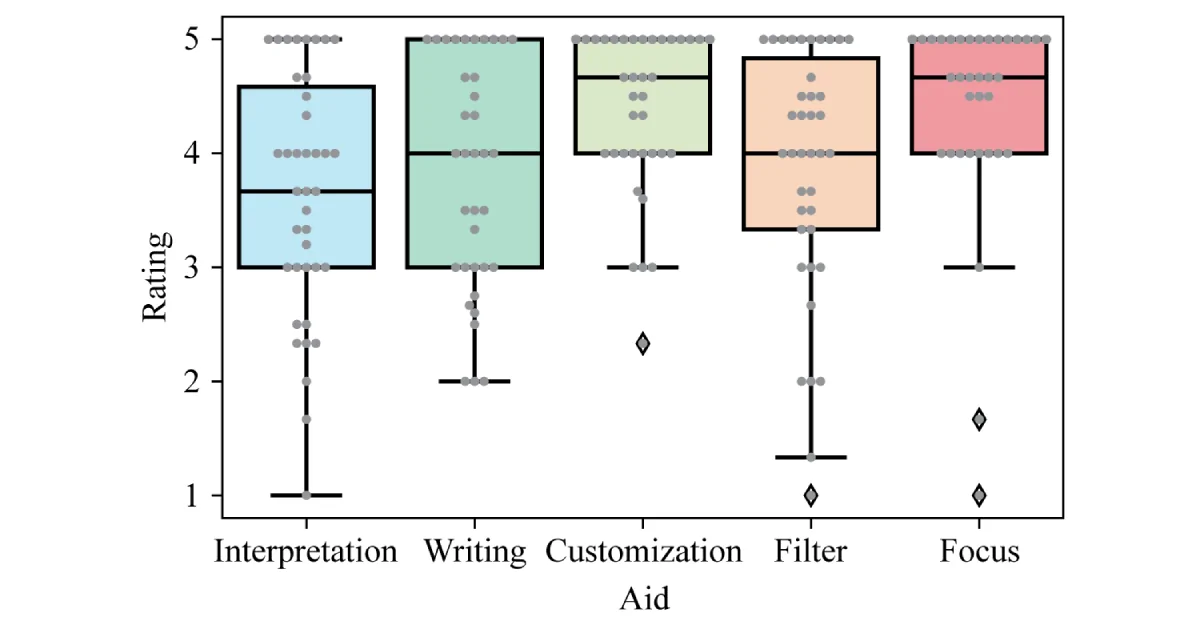
Daily ratings. Aggregate daily ratings of all participants across days of aid use for each of the 5 Social Media Accessibility and Rehabilitation Toolkit (SMART-TBI) extensions.

When evaluating each aid, participants were asked to provide a daily rating on each of the 3 evaluation days. While some participants provided exactly 3 ratings, others provided additional ratings or missed days. In total, 518 ratings were collected throughout the study. Due to the inconsistency in the number of ratings between participants, overall scores were calculated by first generating average scores per participant per aid and then averaging those aggregates to calculate per-aid average ratings. These ratings range from 1 to 5 stars, where 1 star indicates a poor experience and 5 stars indicate a highly positive experience. On average, participants rated their satisfaction with each aid as follows: focus, 4.45 (SD 0.89); customization, 4.41 (SD 0.71); filter, 3.86 (SD 1.09); writing, 3.83 (SD 1.01); and interpretation, 3.65 (SD 1.06). While these scores express an overall positive perception of the aids, there appear to be noteworthy divisions, with the focus and customization aids being rated similarly and most highly, followed by the filter and writing aids, and finally the interpretation aid. These scores are visualized in Figure 3.

#### USE Questionnaire

**Figure 4:**
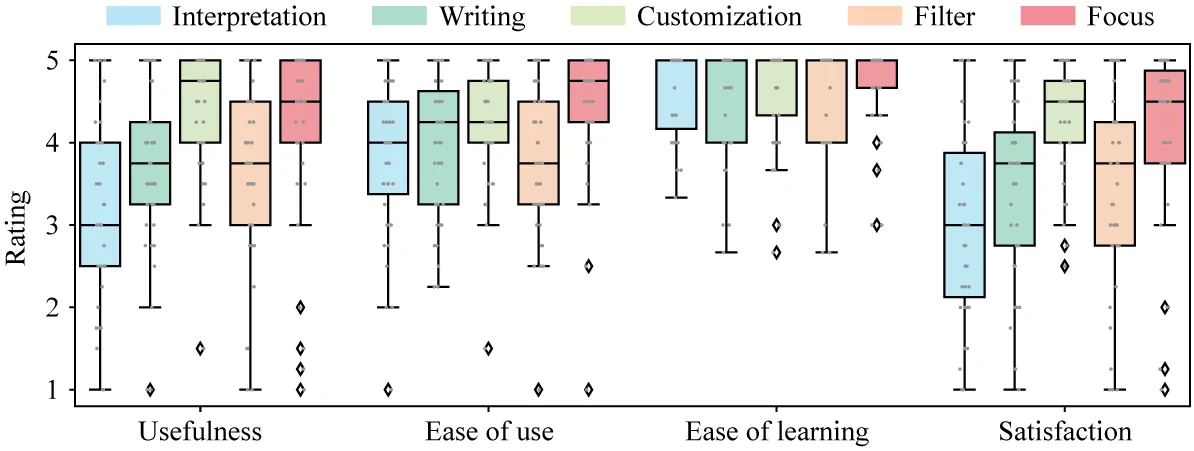
Usefulness, Satisfaction, and Ease of Use (USE) Questionnaire ratings. Participant ratings of usefulness, ease of use, ease of learning, and satisfaction across all 5 Social Media Accessibility and Rehabilitation Toolkit (SMART-TBI) extensions. These subscales are shown in the order in which they are presented within the full scale.

Following each evaluation phase of the study, participants completed the USE Questionnaire. The average ratings per aid in terms of usefulness, ease of use, ease of learning, and satisfaction can be seen in Figure 4. While there is some variation between the daily ratings and holistic postevaluation ratings of satisfaction, we see a similar pattern of perception wherein the customization (mean 4.33, SD 0.68) and focus (mean 4.17, SD 0.98) aids are rated most highly, followed by the filter (mean 3.44, SD 1.19) and writing (mean 3.44, SD 1.05) aids, and finally the interpretation aid (mean 2.97, SD 1.17). The same pattern is also present in participant ratings of aid usefulness and ease of use. There is, however, a noteworthy difference in terms of ease of learning, for which all aids scored 4.56 or higher on average (focus: mean 4.76, SD 0.51; customization: mean 4.64, SD 0.61; interpretation: mean 4.63, SD 0.51; writing: mean 4.57, SD 0.66; filter: mean 4.56, SD 0.73).

#### Likes and Dislikes

##### Overview

Along with the USE Questionnaire, participants also provided open-ended responses regarding aspects of the aids that they most liked and disliked. We collected additional feedback on aid challenges, technical difficulties, and design suggestions, though these data were much sparser, as participants often provided responses such as “none” or “see previous.” Here, we present the most common sentiments shared by participants for each aid.

##### Focus Aid

Participants noted that the focus aid decreased distractions within the Facebook interface, allowed for improved focus, and made their use of Facebook less overwhelming. As one participant stated:

[I] loved how it helped me stay focused on the news feed. [I]t made me feel less overwhelmed and [I] would probably stay on [Facebook] longer due to this.

P30

Participants also expressed that they would have preferred the translucent blurring effect to instead be opaque so that moving content (eg, advertisements) would not be visible. Additionally, participants noted that while it was active, the focus aid made some elements of the interface inaccessible.

##### Filter Aid

The features of the filter aid that participants most liked were the positive filter, the chronological-ordering filter, and the overall ability to control and customize their Facebook experience. The benefits of the positive filter are well illustrated by participant 5, who stated:

I liked knowing that if I was having a bad day and wanted to use the filter...I had the option to engage with Facebook without the fear of encountering any content that could be interpreted as ‘too heavy’ to handle for that day.

This statement captures the need for both limitations on certain types of content and the ability to tailor content based on day-to-day user preferences. Participants also expressed, however, that the filter aid caused lag when scrolling through the newsfeed and that the menu bar covered certain interface elements. The lag experienced while using the aid was caused by information loading and rendering on the web page.

##### Customization Aid

Like the focus aid, participants reported that the customization aid enabled greater control, decreased distractions, and simplified the Facebook interface by removing clutter. These benefits were expressed by participant 12, who stated:

I liked how much [the customization aid] decluttered my feed since I am so easily distracted and overwhelmed by information. I liked that I could choose certain tabs to keep on the left side and hide the rest.

While the customization aid requires users to specify which elements they would like to keep and remove, which could be a cognitive challenge, the result is a more tailored Facebook experience that ultimately could reduce cognitive load and increase the ability to focus on desired content. Depending on the user, this granular selection might be more desirable than the broad blurring of interface elements with the focus aid. The primary negative feedback about the customization aid was the inability to use the aid beyond the Facebook home page, such as blocking advertisements in the Facebook Marketplace.

##### Writing Aid

Participants’ most-liked features were the spelling and grammar checks and the tone and toxicity checks. As stated by participant 27:

I’m glad that [the writing aid] checked the toxicity and tone of the post. It made me think twice about whether or not I wanted to post a comment. I really like that it checked grammar and spelling. There’s nothing worse than bad spelling.

This quote illustrates that participants appreciated the review of their content before posting to limit errors in both communication of intention and technical aspects of writing. While the aid had a positive impact for some participants, others reported aid inaccuracies. For example, participant 19 expressed concern, stating:

It wasn’t very accurate...and it made me anxious—if I think I can trust something like that I might let my guard down and depend on it. When I find it’s not accurate, it’s disturbing.

##### Interpretation Aid

Participants reported that the interpretation aid allowed for easier understanding of posts by clarifying sentiment and summarizing content. Participant 27 reported a positive experience with the aid, stating:

I really liked that it colored the text. I found myself not reading the red text at all and skimming through the white text. It really made the whole experience more positive and more enjoyable.

Not all participants felt they needed the aid. Participant 29 said:

[The interpretation aid] wasn’t something that I would use, but it is also something that I don’t need. For those who struggle with perception it could be useful.

Some participants reported disagreeing with the interpretations. As with the writing aid, participant 19 said, “I had a hard time trusting [the interpretation aid] because it didn’t always agree with my interpretations of what was positive or negative.”

### Poststudy Evaluation

#### Overview

Participants were asked 3 questions at the conclusion of the study about their experience using the aids and their overall favorite aid. The following themes emerged from these data.

#### Overall Positive Experience Using the Aids

A total of 95% (37/39) of participants reported positive experiences using the aids. Participants appreciated the ease of learning and using the aids, with participant 24 stating:

I had a very good experience using the aids. They were all easy to learn how to use and didn’t have too many kinks. I definitely felt like some were more specific to the symptoms I struggle with than others.

The aids were noted to enhance engagement and enjoyment on Facebook, as evidenced by participants’ comments like, “I absolutely loved the aids. I loved that they kept me more focused and less distracted. I was able to enjoy Facebook more” (P27) and “It was very helpful, and I feel like I would use Facebook more” (P35). Participants also emphasized the aids’ user-friendliness, noting, “All aids were easy to use” (P22) and “The tools were really useful based on my needs” (P1), though some aids were easier to use than others. Additionally, the aids were recognized for their potential value to individuals with TBI, with one participant stating, “I believe the aids are very helpful for the Brain Injury Community” (P37), and another stating, “They each could have value depending on the user’s needs” (P22). Overall, the aids were well received, contributing to increased focus, enjoyment, and perceived value among participants.

A total of 21% (8/39) of participants also reported specific negative experiences while using the aids. Technical difficulties were a common issue, as evidenced by participant comments such as, “Sometimes [the aids] didn’t work well enough to make them worth using” (P19) and “I found a lot of errors” (P14). The interpretation aid was particularly noted to have technical challenges: “The interpretation aid was glitchy. I think it could be easier if it was working. Ideally, it could be really great” (P8). Some participants questioned the usefulness of certain aids, with one stating, “Some of the aid[s] I was not able to really understand how it would be beneficial for someone with TBI” (P14). Technological difficulties with the equipment were also mentioned:

The aids...I don’t know if it was the Chromebook that was making [sic], or the debugging of the aid, but the Facebook responses would come out slower or slow. It would be a long time waiting for something to happen while using the aid. The waiting was frustrating. Nowadays, everyone wants things to be done very quick. Waiting just for a Facebook post or feed, sometimes it’s not worth the wait. A lot of times, I would just close the window because I thought it was crashing, and would have to close and restart.

P1

Issues with accuracy were highlighted, with one participant noting, “The writing tool was not as accurate, and I found it frustrating when there were so many technology glitches” (P2). Lastly, some participants raised concerns about the potential harm of using AI for functions such as sentiment analysis:

Everything else has the potential to be harmful for users, especially for those who want to engage in self-expression and just want help with navigating online interactions.

P5

#### Favorite Aids: Focus, Filter, and Facebook Customization

##### Overview

Participants were asked to name their favorite overall aid from the study. The focus, filter, and Facebook customization aids emerged as their favorites, with some participants ranking more than 1 aid as their favorite.

##### Focus Aid

A total of 49% (19/39) of participants appreciated the focus aid’s straightforward and easy-to-use interface, noting that it significantly reduced distractions and kept the user interface clutter-free, thereby allowing for a more focused Facebook experience. For instance, participant 3 shared:

I liked everything! Like OMG, yesterday I was watching some cooking stuff, and the fact there is no distraction, nothing on the left that is taking my attention. Just to focus on one thing, it brought some peace of mind, less anxious. Yes, I love the focus mode.

Similarly, participant 6 said:

I loved this one because when you’re on Facebook, and all the apps disappear, then it’s less distraction. And you’re more focused on what you’re doing.

The focus aid also helped reduce feelings of being overwhelmed, as highlighted by participant 10, who said:

Focus Mode was my favorite. I felt less overwhelmed using Facebook and could pay more attention to what I was looking at, it felt easier to try to and write something without so much to pay attention to.

Participant 7 echoed this sentiment, stating, “I was able to focus on what I was doing, and ignore the ‘visual noise’.”

##### Filter Aid

A total of 41% (16/39) of participants reported various features they enjoyed about the filter aid. They appreciated the ability to control what they see, such as filtering by positive posts: “I really enjoyed the ability to block negative posts” (P37) and to only show positive posts (P32 and P28). Others valued filtering by the most recent posts: “I like having the ability to see posts in chronological order!” (P11). Participants also noted the aid’s ability to prioritize important posts over advertisements and irrelevant content. For example, participant 19 remarked:

I really liked the one that filtered my posts to where the pertinent ones were the ones that would show up—I would use this all the time. I get too easily sucked into ads and distracted by them, and it seems most of my feed is ads—it’s not worth wading through all of them to find the more important posts. I don’t like re-reading the same posts over and over either, so to have them in chronological order keeps them from repeating and saves my energy.

##### Facebook Customization Aid

A total of 36% (14/39) of participants chose the Facebook customization aid as a favorite. One participant noted:

I liked the Facebook Customization aid the best in that it allowed me to turn off the various panels of FB [sic] which increased my desire to interact with FB [sic].

P36

Others noted that Facebook can be cluttered, and the aid helped with this problem:

Facebook can feel so cluttered with all the options of things to click on, that I rarely even click the options I like. To have the few options I would want to click on made Facebook look so much cleaner, not cluttered, and tailored to exactly what I would use.

P15

Participants appreciated the flexibility and simplicity of the aid and that it made navigating the Facebook page less busy (P29 and P22). Others noted that decluttering the page, similar to the Focus aid, helped minimize distractions, allowing them to “be able to focus on the content people posted rather than getting distracted” (P26). Participants also appreciated that the aid minimized unwanted content that could be potentially emotionally triggering:

The Facebook Customization was a game changer. It helped me a lot on my bad days, when I was feeling really emotional. I could just display exactly what I was able to view on that day.

P2

During the final poststudy survey, participants reported which of the aids they would use in daily life if given the opportunity. Of the 39 participants, 21 (54%) chose the filter and focus aids, 19 (49%) chose the customization aid, 11 (28%) chose the writing aid, and 9 (23%) chose the interpretation aid. These results largely comport with the other metrics we have reviewed; however, the filter aid was endorsed by more participants than expected. This misalignment with our other collected measures might be due to the fact that the aid endorsement questions asked which aids participants would like to use rather than which they thought worked the best. Participants may have believed the filter aid would be highly useful after further development, but that its current form did not quite meet that potential. A visualization of per-aid endorsement counts is provided in Multimedia Appendix 4.

#### Impact on Participants

**Figure 5:**
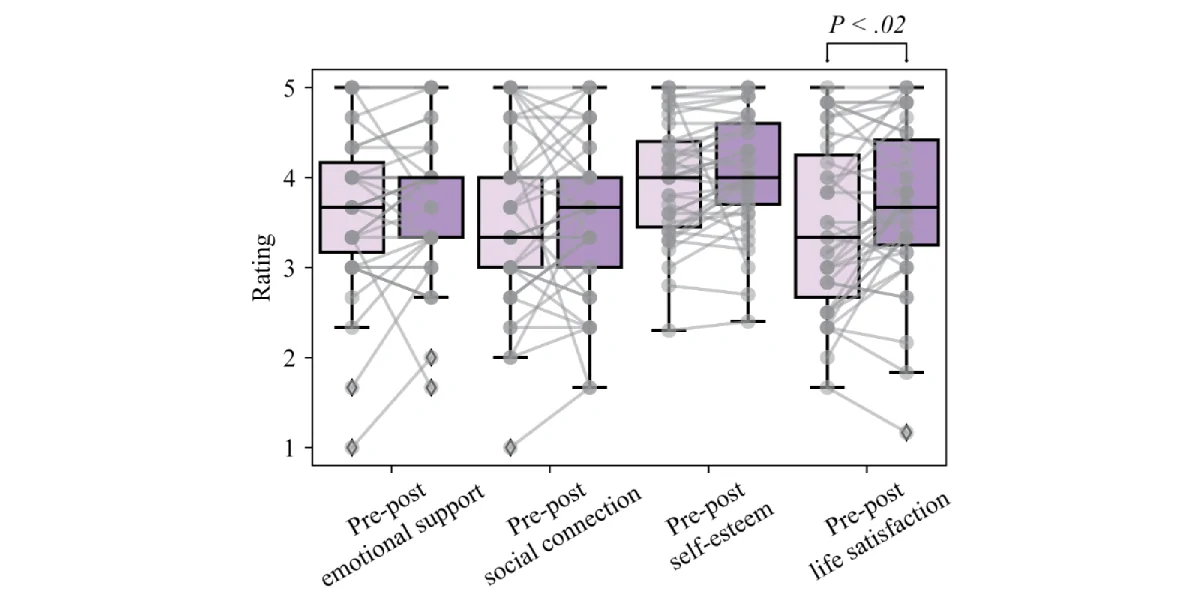
Pre-post study metrics. Participants reported their perception of emotional support, social connection, self-esteem, and life satisfaction prior to and following their engagement in this study. A statistically significant increase was found between participants’ life satisfaction before and after participation.

Next, we attempted to understand how participants may have been impacted by each of our aids. Toward this effort, we conducted a pre-post study analysis to understand whether participants reported changes in feelings of emotional support, social connection, self-esteem, and life satisfaction (Figure 5). Further, 1-tailed paired-samples *t* tests with Bonferroni correction showed a statistically significant increase in participant ratings of satisfaction with life (*P*=.02) and nonsignificant increases across the remaining 3 metrics following the use of our aids. A full listing of the relevant statistics from these tests can be seen in Table 2.

**Table 2:** Paired-samples *t* test statistics. The study used 1-tailed paired-samples *t* tests to assess statistical significance in pre-post differences between participants’ reported perceptions of emotional support, social connection, self-esteem, and life satisfaction. A statistically significant increase was only found in perceptions of life satisfaction.

Outcome	Pretest, mean (SD)	Posttest, mean (SD)	*t* test (*df*)	Corrected *P* value	95% CI	Cohen *d*
Emotional support	3.684 (0.88)	3.761 (0.80)	−0.851 (38)	.80	−∞ to 0.075	−0.091
Social connection	3.496 (0.95)	3.547 (0.88)	−0.406 (38)	>.99	−∞ to 0.162	−0.055
Self-esteem	3.972 (0.66)	4.072 (0.64)	−1.441 (38)	.32	−∞ to 0.017	−0.153
Life satisfaction	3.449 (0.94)	3.705 (0.88)	−2.666 (38)	.02	−∞ to −0.094	−0.278

## Discussion

### Principal Findings

Participants with moderate to severe TBI have cognitive, sensorimotor, and communication challenges that can make social media engagement difficult or unmanageable. Our study illustrates that many of these challenges can be addressed through proper technological supports. Through this work, we have (1) demonstrated the feasibility of a longitudinal study design to examine the social media use of individuals with TBI, (2) gained novel insights into the social media experience of users with TBI and related design implications, and (3) identified new directions for researchers and developers working to promote online social connection among users with TBI.

### SMART-TBI Perceptions

Through our results, we have shown that, on average, all of our aids were regarded positively by participants across all metrics. The filter, focus, and customization aids were rated by participants as their favorite aids and the aids they would have most liked to use beyond the study. These results are consistent with participants’ reported sensory and cognitive challenges, as all 3 aids allowed users to configure what was displayed onscreen.

The writing and interpretation aids were regarded less positively overall. This result was unsurprising given that social challenges can be more complex and multidimensional than sensory limitations and, hence, more difficult to overcome with a technical solution. The sentiment analyses used in these aids also yielded inconsistent results, which meant they were regarded by some as untrustworthy. Participant perception may then be more reflective of our specific implementation than the core ideas the aids represent. Across daily ratings and all USE metrics, the writing and interpretation aids were consistently rated the lowest. Future implementations may be able to provide the aids’ intended utility and be perceived as highly useful.

It is noteworthy that the findings did not apply to all participants, as some found the writing and interpretation aids very helpful in their current form. This variability in findings is common in TBI due to the heterogeneity across this clinical population in strengths and limitations, previous experience, and goals. It is expected that some users will benefit from certain aids, while others will not. If implemented in a commercially available product, the problems associated with these aids would likely be addressed, and users would be able to use them fully. Even with such an implementation, however, it is important to consider the impact that bias, subtle misclassifications, and overreliance may have on users. While our study did not capture the nuance of these problems, they could introduce new challenges to social media users with TBI. Through proper research and development, these challenges may also be mitigated, but further evaluation will be needed.

### Accessibility Design Insights

Beyond the aids themselves, we also identified the following three major insights from our study results: (1) participants preferred aids that assisted with sensory and cognitive challenges over social ones, (2) participants would like to tailor their social media experience to their specific needs, and (3) simple solutions to TBI challenges were preferred over complex ones.

Participants preferred aids that assist with TBI-related sensory and cognitive challenges over social ones. These findings are consistent with Zhao et al [48], who proposed that social deficits are difficult to recognize in oneself and therefore make an individual less likely to believe they may need related assistance. While the focus, filter, and customization aids all allowed users to make their Facebook experience more manageable through selection of interface elements and newsfeed content, the writing and interpretation aids attempted to compensate for social challenges by interpreting content for the user. While most interface and content changes are objective and clear, interpretations are subjective and more difficult to assess in terms of accuracy. When participants assess that some interpretations are not accurate, a challenging judgment in its own right, they may lose a sense of trust in all interpretations. As a result, these types of aids may be seen as fraught compared with those that address cognitive and sensory challenges. Additional validation will therefore be needed to improve these aids and understand the implications of their use prior to widespread implementation on social media platforms.

Consistent with the variability among people with TBI, participants appreciated the ability to tailor their social media interface to fit their needs. The ability to customize aids is essential in TBI to allow users the freedom to activate features as needed or select a baseline set of features that fit their needs. This point was well illustrated by participants’ positive ratings of the filter and customization aids, which allow users to explicitly select which interface elements appear on their screen and what content appears in their newsfeed. Allowing users to choose specific accessibility features ensures that benefits are fully realized without making the resulting experience needlessly cumbersome.

While user choice allows specific needs to be met, it can also generate greater complexity to the detriment of a user’s experience. If we consider, for example, the focus and writing aids, they each require different levels of effort from users. In the case of the focus aid, users simply activate the aid to blur the left and right panels of the Facebook interface. With the writing aid, however, participants activate the aid and then follow several steps while writing a post. While both of these aids have unique advantages and disadvantages, the writing aid adds much greater complexity to the baseline use of Facebook than does the focus aid. While we cannot disentangle the utility of a given aid from its cognitive demand, our results do suggest that participants prefer simpler aids (ie, the focus, filter, and customization aids) to more complex ones (ie, the interpretation and writing aids).

### Limitations and Future Work

While this work shows great promise for the use of social media aids, some challenges have remained unaddressed. To begin, we had intended for participants to use our aids in their daily Facebook use as would occur naturally. However, it is clear from participants’ daily ratings that participants would experiment with the aids to understand their limitations. For example, some participants wrote draft posts that included highly positive, negative, or subjective sentiments to evaluate the accuracy of the writing aid’s sentiment analysis. While these participants’ curiosity is understandable, these interactions illustrate the potential limitations inherent to an in-the-wild study. Additionally, we found that some participants had difficulty remembering whether they had used Facebook on a given day, leading to missed or duplicated participation. This difficulty is understandable and to be expected among participants who experience memory challenges. To address this challenge, experimenters provided friendly reminders to participants about daily participation, but challenges persisted. In the future, we believe that a usage indicator would be helpful for users to determine whether they had already participated on a given day. Such an indicator could take the form of a small overlay dashboard that contains an icon to signal whether the participant had completed their use for the day.

Furthermore, we saw that not all challenges participants reported were addressed by our aids. In particular, we expect that the writing and interpretation aids can be refined for a better overall user experience by improving their sentiment analysis features. Additionally, while the filter aid was positively regarded, we believe there are other filter options we can provide to users so they can fully tailor their display to their specific preferences. Beyond our existing aids, we also believe there are opportunities for new supports that can assist users on other social media platforms. While the current aids were developed specifically for Facebook, there may also be opportunities to apply these concepts to other platforms. Some supports, like the focus aid, may need to be tailored to the specific interface elements of a given platform, but others, like the filter aid, could easily be translated to platforms that use a feed. As social media evolves over time, similar adaptations may be needed, but, provided the core features of social media remain (eg, feed, posting, comments, and advertisements), the benefits these aids provide should continue to be relevant to individuals with TBI. Examining how these aids can be integrated into other platforms and exploring their potential impact on other user populations would substantially widen their impact.

Beyond the development and use of the aids, it is worth noting that the compensation participants received (ie, monetary compensation for participation and the gifted Chromebook) could have skewed their perceptions. While many participants did provide critical feedback about the aids, daily use and life satisfaction ratings could have been skewed by monetary gains and the novelty of using a new device. Additionally, while our results show a statistically significant increase in life satisfaction between the beginning and end of our study, we do not see a significant increase in emotional support, social connection, or self-esteem. Given that changes of this nature may require greater exposure to our aids, further evaluation is needed to understand whether the use of such aids can achieve these outcomes.

Finally, it is important to consider the generalizability of our results. To be considered for our study, participants needed to be active users of Facebook who were willing to partake in a technology-evaluation study. Our results may therefore be limited to users with higher digital literacy and more social media experience than the average individual with TBI. Future work may be able to bridge this gap by evaluating such aids with a broader population of users with TBI, including those with more severe cognitive impairments or anosognosia. Additionally, our study does not use a comparison group or randomized controlled design. This step could be taken in similar future studies to clearly contextualize the perceptions of the developed aids and the broader impacts these aids have on participant well-being.

### Conclusion

Users with TBI experience challenges related to sensory perception, cognition, communication, and social interaction. While social media platforms like Facebook can provide an outlet for users to maintain connections post injury, these platforms must provide TBI-specific accessibility features so that users may enjoy the full benefits of their social media experience. To that end, we created 5 digital aids that allowed users to modify their Facebook user interface and collected feedback on use of the aids in a longitudinal study with 39 Facebook users with TBI. While participants had varying needs and preferences for the aid extensions, overall, they had positive ratings for the aids’ accessibility features. These findings suggest that aids like these have the potential to improve the social media experience of users with TBI.

## Appendix

Multimedia Appendix 1 Demographic and injury characteristics of the 39 participants included in the longitudinal study. The “other” cause of traumatic brain injury includes highly specific circumstances that may otherwise identify participants. Although motorcycle collisions (MCCs) are encompassed within motor vehicle accidents (MVAs), they are categorized separately due to the unique risks and injury patterns associated with motorcycle-related injuries.

Multimedia Appendix 2 Questionnaires used during prestudy interviews and aid evaluation surveys. The former allowed the research team to assess participants’ traumatic brain injury (TBI) and social media use histories, while the latter enabled evaluation of each Social Media Accessibility and Rehabilitation Toolkit (SMART-TBI) extension.

Multimedia Appendix 3 Social media platform use—counts of participants who reported using major social media platforms.

Multimedia Appendix 4 Counts of participants who reported they would use each aid in their daily lives if given the opportunity. These data were collected during the poststudy survey, although additional endorsements were also captured during the poststudy interview.

## Abbreviations

GCS: Glasgow Coma Scale
HIPAA: Health Insurance Portability and Accountability Act
IRB: institutional review board
LOC: loss of consciousness
PTA: posttraumatic amnesia
SMART-TBI: Social Media Accessibility and Rehabilitation Toolkit
TBI: traumatic brain injury
USE: Usefulness, Satisfaction, and Ease of Use

## References

[1] Mazaux JM, Masson F, Levin HS, Alaoui P, Maurette P, Barat M (1997). Long-term neuropsychological outcome and loss of social autonomy after traumatic brain injury. Arch Phys Med Rehabil.

[2] Sohlberg MM, Hamilton J, Turkstra L (2023). Transforming Cognitive Rehabilitation: Effective Instructional Methods.

[3] Togher L, McDonald S, Coelho CA, Byom L (2013). Cognitive communication disability following TBI: examining discourse, pragmatics, behaviour and executive function. Social and Communication Disorders Following Traumatic Brain Injury.

[4] Brunner M, Palmer S, Togher L, Hemsley B (2019). 'I kind of figured it out': the views and experiences of people with traumatic brain injury (TBI) in using social media-self-determination for participation and inclusion online. Int J Lang Commun Disord.

[5] Morrow EL, Zhao F, Turkstra L, Toma C, Mutlu B, Duff MC (2021). Computer-mediated communication in adults with and without moderate-to-severe traumatic brain injury: survey of social media use. JMIR Rehabil Assist Technol.

[6] Lim H, Kakonge L, Hu Y, Turkstra L, Duff M, Toma C, Mutlu B (2023). So, I can feel normal: participatory design for accessible social media sites for individuals with traumatic brain injury.

[7] Flynn MA, Rigon A, Kornfield R, Mutlu B, Duff MC, Turkstra LS (2019). Characterizing computer-mediated communication, friendship, and social participation in adults with traumatic brain injury. Brain Inj.

[8] Hu Y, Lim H, Kakonge L, Mitchell J, Johnson H, Turkstra L, Duff M, Toma C, Mutlu B (2024). SMART-TBI: design and evaluation of the social media accessibility and rehabilitation toolkit for users with traumatic brain injury.

[9] Byom L, O'Neil-Pirozzi TM, Lemoncello R, MacDonald S, Meulenbroek P, Ness B, Sohlberg MM, Academy of Neurologic Communication Disorders Traumatic Brain Injury Writing Committee (2020). Social communication following adult traumatic brain injury: a scoping review of theoretical models. Am J Speech Lang Pathol.

[10] Tsai Y-C, Liu C-J, Huang H-C, Lin J-H, Chen P-Y, Su Y-K, Chen C-T, Chiu H-Y (2021). A meta-analysis of dynamic prevalence of cognitive deficits in the acute, subacute, and chronic phases after traumatic brain Injury. J Neurosci Nurs.

[11] Diamond A (2013). Executive functions. Annu Rev Psychol.

[12] Lannoo E, Colardyn F, Vandekerckhove T, De Deyne C, De Soete G, Jannes C (1998). Subjective complaints versus neuropsychological test performance after moderate to severe head injury. Acta Neurochir (Wien).

[13] Rochat L, Beni C, Annoni J, Vuadens P, Van der Linden M (2013). How inhibition relates to impulsivity after moderate to severe traumatic brain injury. J Int Neuropsychol Soc.

[14] Cassel A, McDonald S, Kelly M, Togher L (2019). Learning from the minds of others: a review of social cognition treatments and their relevance to traumatic brain injury. Neuropsychol Rehabil.

[15] Allain P, Hamon M, Saoût V, Verny C, Dinomais M, Besnard J (2019). Theory of mind impairments highlighted with an ecological performance-based test indicate behavioral executive deficits in traumatic brain injury. Front Neurol.

[16] Spikman JM, Timmerman ME, Milders MV, Veenstra WS, van der Naalt J (2012). Social cognition impairments in relation to general cognitive deficits, injury severity, and prefrontal lesions in traumatic brain injury patients. J Neurotrauma.

[17] Channon S, Crawford S (2010). Mentalising and social problem-solving after brain injury. Neuropsychol Rehabil.

[18] McDonald S (2013). Impairments in social cognition following severe traumatic brain injury. J Int Neuropsychol Soc.

[19] Bibby H, McDonald S (2005). Theory of mind after traumatic brain injury. Neuropsychologia.

[20] Bosco FM, Angeleri R, Sacco K, Bara BG (2015). Explaining pragmatic performance in traumatic brain injury: a process perspective on communicative errors. Int J Lang Commun Disord.

[21] Turkstra LS, Norman RS, Mutlu B, Duff MC (2018). Impaired theory of mind in adults with traumatic brain injury: a replication and extension of findings. Neuropsychologia.

[22] Vascello MGF, Pizzighello S, Spada MS, Martinuzzi A, Dalmaso M (2024). Social face processing in chronic severe traumatic brain injury: altered decoding of emotions and mental states but preserved gaze cueing of attention. Neuropsychologia.

[23] Rodríguez-Rajo P, García-Rudolph A, Sánchez-Carrión R, Aparicio-López C, Enseñat-Cantallops A, García-Molina A (2022). Social and nonsocial cognition: are they linked? A study on patients with moderate-to-severe traumatic brain injury. Appl Neuropsychol Adult.

[24] Byom L, Turkstra LS (2017). Cognitive task demands and discourse performance after traumatic brain injury. Int J Lang Commun Disord.

[25] Filipčíková M, Quang H, Cassel A, Darke L, Wilson E, Wearne T, Rosenberg H, McDonald S (2024). Exploring neuropsychological underpinnings of poor communication after traumatic brain injury: the role of apathy, disinhibition and social cognition. Int J Lang Commun Disord.

[26] Westerhof-Evers HJ, Fasotti L, van der Naalt J, Spikman JM (2019). Participation after traumatic brain injury: the surplus value of social cognition tests beyond measures for executive functioning and dysexecutive behavior in a statistical prediction model. Brain Inj.

[27] Togher L, Wiseman-Hakes C, Douglas J, Stergiou-Kita M, Ponsford J, Teasell R, Bayley M, Turkstra LS, INCOG Expert Panel (2014). INCOG recommendations for management of cognition following traumatic brain injury, part IV: cognitive communication. J Head Trauma Rehabil.

[28] VanSolkema M, McCann C, Barker-Collo S, Foster A (2020). Attention and communication following TBI: making the connection through a meta-narrative systematic review. Neuropsychol Rev.

[29] Wise EK, Mathews-Dalton C, Dikmen S, Temkin N, Machamer J, Bell K, Powell JM (2010). Impact of traumatic brain injury on participation in leisure activities. Arch Phys Med Rehabil.

[30] Jourdan C, Bayen E, Pradat-Diehl P, Ghout I, Darnoux E, Azerad S, Vallat-Azouvi C, Charanton J, Aegerter P, Ruet A, Azouvi P (2016). A comprehensive picture of 4-year outcome of severe brain injuries. Results from the PariS-TBI study. Ann Phys Rehabil Med.

[31] Hoofien D, Gilboa A, Vakil E, Donovick PJ (2001). Traumatic brain injury (TBI) 10-20 years later: a comprehensive outcome study of psychiatric symptomatology, cognitive abilities and psychosocial functioning. Brain Inj.

[32] Engberg AW, Teasdale TW (2004). Psychosocial outcome following traumatic brain injury in adults: a long-term population-based follow-up. Brain Inj.

[33] Doig E, Fleming J, Tooth L (2001). Patterns of community integration 2-5 years post-discharge from brain injury rehabilitation. Brain Inj.

[34] McDonald S, Genova H (2021). The effect of severe traumatic brain injury on social cognition, emotion regulation, and mood. Handb Clin Neurol.

[35] Hu Y, Lim H, Johnson HL, O'Shaughnessy J, Kakonge L, Turkstra L, Duff M, Toma C, Mutlu B (2023). Investigating day-to-day experiences with conversational agents by users with traumatic brain injury.

[36] Dahlberg C, Hawley L, Morey C, Newman J, Cusick CP, Harrison-Felix C (2006). Social communication skills in persons with post-acute traumatic brain injury: three perspectives. Brain Inj.

[37] MacDonald S (2017). Introducing the model of cognitive-communication competence: a model to guide evidence-based communication interventions after brain injury. Brain Inj.

[38] Brunner M, Palmer S, Togher L, Dann S, Hemsley B (2019). “If I knew what I was doing on Twitter then I would use it more”: Twitter experiences and networks of people with traumatic brain injury (TBI). Brain Impairment.

[39] Toma CL, Hwang J, Kakonge L, Morrow EL, Turkstra LS, Mutlu B, Duff MC (2024). Does Facebook use provide social benefits to adults with traumatic brain injury?. Cyberpsychol Behav Soc Netw.

[40] Brunner M, Hemsley B, Palmer S, Dann S, Togher L (2015). Review of the literature on the use of social media by people with traumatic brain injury (TBI). Disabil Rehabil.

[41] Tsaousides T, Matsuzawa Y, Lebowitz M (2011). Familiarity and prevalence of facebook use for social networking among individuals with traumatic brain injury. Brain Inj.

[42] Feuston JL, Marshall-Fricker CG, Piper AM (2017). The social lives of individuals with traumatic brain injury.

[43] Brunner M, Palmer S, Togher L, Dann S, Hemsley B (2019). Content analysis of tweets by people with traumatic brain injury (TBI): implications for rehabilitation and social media goals.

[44] Clough S, Morrow E, Mutlu B, Turkstra L, Duff MC (2023). Emotion recognition of faces and emoji in individuals with moderate-severe traumatic brain injury. Brain Inj.

[45] Kaplan AM, Haenlein M (2010). Users of the world, unite! The challenges and opportunities of social media. Bus Horiz.

[46] Brunner M, Rietdijk R, Avramovic P, Power E, Miao M, Rushworth N, MacLean L, Brookes A, Togher L (2023). Developing social-ABI-lity: an online course to support safe use of social media for connection after acquired brain injury. Am J Speech Lang Pathol.

[47] Ahmadi R, Lim H, Mutlu B, Duff M, Toma C, Turkstra L (2022). Facebook experiences of users with traumatic brain injury: a think-aloud study. JMIR Rehabil Assist Technol.

[48] Zhao F, Lim H, Morrow EL, Turkstra LS, Duff MC, Mutlu B (2022). Designing evidence-based support aids for social media access for individuals with moderate-severe traumatic brain injury: a preliminary acceptability study. Front Digit Health.

[49] IBM Watson Natural Language Understanding. IBM.

[50] Check your texts online: grammar, spelling, and readability. TextGears.

[51] Enabling online conversations. Perspective.

[52] Duff MC, Morrow EL, Edwards M, McCurdy R, Clough S, Patel N, Walsh K, Covington NV (2022). The value of patient registries to advance basic and translational research in the area of traumatic brain injury. Front Behav Neurosci.

[53] Malec JF, Brown AW, Leibson CL, Flaada JT, Mandrekar JN, Diehl NN, Perkins PK (2007). The Mayo classification system for traumatic brain injury severity. J Neurotrauma.

[54] Lund AM (2001). Measuring usability with the use questionnaire12. Usability interface.

[55] Ledbetter AM (2009). Measuring online communication attitude: Instrument development and validation. Commun Monogr.

[56] Rosenberg M (1965). Society and the Adolescent Self-Image.

[57] Diener E (1985). Satisfaction with life scale. (J Pers Assess.

[58] Braun V, Clarke V (2008). Using thematic analysis in psychology. Qual Res Psychol.

[59] Braun V, Clarke V (2022). Conceptual and design thinking for thematic analysis. Qual Psychol.

[60] (2018). Dedoose version 8.0.35: web application for managing, analyzing, and presenting qualitative and mixed method research data. Dedoose.

