# Supplementary material for: Digital Aids for Social Media in Adults With Traumatic Brain Injury: Longitudinal Evaluation Study
Source: JMIR Rehabil Assist Technol. 2026 Aug 26;13:e83639. doi: 10.2196/83639 (PMC13559156; doi:10.2196/83639)
Supplement: Supplementary file 1 [file rehab_v13i1e83639_app2.pdf]

Pre-Study Interview Questions:

- Has anything changed about your Facebook use since your injury?
- Have you ever used apps or tools to assist you in overcoming any of your symptoms while using the internet?
- Do you currently use any of those technologies? And if so, what do you like about them?
- How often do you use social media?
- How often do you use Facebook?
- What other social media platforms do you use, if any?
- Do you have any concerns about using social media?
- What do you like about using social media?

Aid Evaluation Questions

- What did you like about the [*aid name*] aid?
- What did you dislike about the [*aid name*] aid?
- Describe any technical difficulties you had while using the [*aid name*] aid. If there were none, write "None."
- What other challenges did you face while using the [*aid name*] aid, if any?
- Is there any way we could have made the [*aid name*] aid better? If yes, please explain.
